# Supplementary material for: Determination of Three Typical Metabolites of Pyrethroid Pesticides in Tea Using a Modified QuEChERS Sample Preparation by Ultra-High Performance Liquid Chromatography Tandem Mass Spectrometry
Source: Foods. 2021 Jan 18;10(1):189. doi: 10.3390/foods10010189 (PMC7831930; doi:10.3390/foods10010189)
Supplement: Supplementary file 1 [file foods-10-00189-s001.pdf]

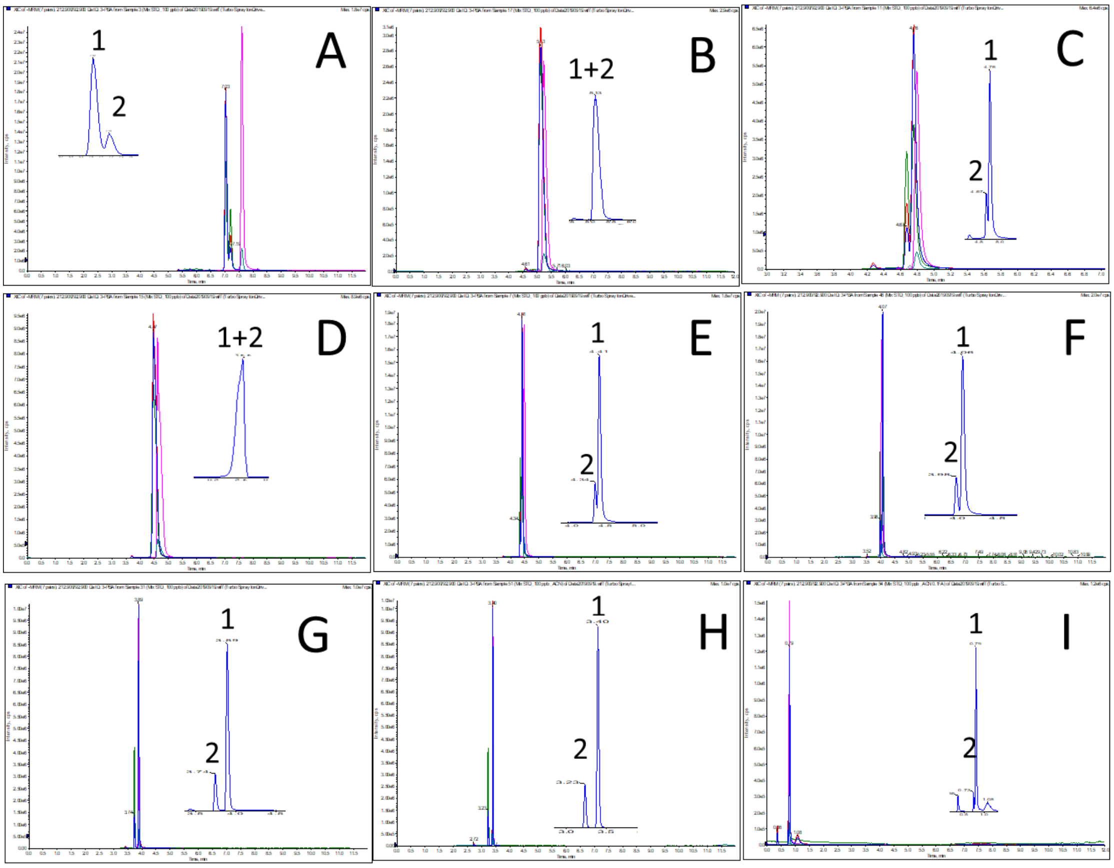

Figure S1. UHPLC-MS/MS chromatograms of 3 target compounds and internal standard 2-PBA (peak 1 means 3-PBA and peak 2 means 2-PBA) obtained from Zorbax Eclipse Plus C18(3.0 *150 mm 1.8 um) (A), Zorbax SB-Aq (2.1 *100 mm 1.8 um) (B), Acquity UHPLC HSS T3 (2.1 *100 mm 1.8 um) (C), Poroshell 120 EC-C18 (2.1 *100 mm 2.7 um) (D), Zorbax Eclipse XDB-C18(3.0 *150 mm 1.8 um) (E), Kinetex C18 (2.1 *50 mm 2.6 um) (F), Kinetex C18 (2.1 *100 mm 2.6 um) under the mobile phase A water and phase B methanol (G), and phase B acetonitrile (H), and phase water Kinetex C18 containing 0.1% formic acid (I).

Note, gradient elution: 0-1min 5% B; 1-3 min 95% B; 3-10 min 95% B, 10-10.5 min 5% B;10.5-12 min 5% B
